# Description and process evaluation of pharmacists' interventions in a pharmacist-led information technology-enabled multicentre cluster randomised controlled trial for reducing medication errors in general practice (PINCER trial)

**DOI:** 10.1111/ijpp.12039

**Published:** 2013-05-30

**Authors:** Rachel Howard, Sarah Rodgers, Anthony J Avery, Aziz Sheikh

**Affiliations:** a School of Pharmacy, University of ReadingReading, UK; b Division of Primary Care, University of Nottingham, University ParkNottingham, UK; c Division of Primary Care, Nottingham University Medical School, Queens Medical CentreNottingham, UK; d Centre for Population Health Sciences, The University of Edinburgh Medical SchoolEdinburgh, UK

**Keywords:** medicines management, monitoring, patient safety, pharmacist, prescribing, primary care

## Abstract

**Objective:**

To undertake a process evaluation of pharmacists' recommendations arising in the context of a complex IT-enabled pharmacist-delivered randomised controlled trial (PINCER trial) to reduce the risk of hazardous medicines management in general practices.

**Methods:**

PINCER pharmacists manually recorded patients' demographics, details of interventions recommended, actions undertaken by practice staff and time taken to manage individual cases of hazardous medicines management. Data were coded, double-entered into SPSS version 15 and then summarised using percentages for categorical data (with 95% confidence interval (CI)) and, as appropriate, means (± standard deviation) or medians (interquartile range) for continuous data.

**Key findings:**

Pharmacists spent a median of 20 min (interquartile range 10, 30) reviewing medical records, recommending interventions and completing actions in each case of hazardous medicines management. Pharmacists judged 72% (95% CI 70, 74; 1463/2026) of cases of hazardous medicines management to be clinically relevant. Pharmacists recommended 2105 interventions in 74% (95% CI 73, 76; 1516/2038) of cases and 1685 actions were taken in 61% (95% CI 59, 63; 1246/2038) of cases; 66% (95% CI 64, 68; 1383/2105) of interventions recommended by pharmacists were completed and 5% (95% CI 4, 6; 104/2105) of recommendations were accepted by general practitioners (GPs), but not completed at the end of the pharmacists' placement; the remaining recommendations were rejected or considered not relevant by GPs.

**Conclusions:**

The outcome measures were used to target pharmacist activity in general practice towards patients at risk from hazardous medicines management. Recommendations from trained PINCER pharmacists were found to be broadly acceptable to GPs and led to ameliorative action in the majority of cases. It seems likely that the approach used by the PINCER pharmacists could be employed by other practice pharmacists following appropriate training.

## Introduction

An estimated 16.5% of patients in primary (ambulatory) care are estimated to experience preventable adverse drug events.[1] Preventable adverse drug events are associated with hazardous medicines management and their incidence can therefore potentially be reduced by improving the safety of prescribing and monitoring in primary care. This patient safety role of pharmacists in UK hospital settings is well established with pharmacists' interventions being widely accepted as improving patient care.[2–4] However, the role of pharmacists in improving patient safety in general practice is in contrast far less well established with conflicting evidence from studies. Our systematic review and meta-analysis found weak evidence that pharmacist interventions in primary care could reduce hospital admissions.[5] Subsequent studies, however, found that pharmacist-led medication reviews have either no effect on hospital admissions[6] or increase hospital admissions.[7]

The PINCER trial was a cluster randomised controlled trial which demonstrated that pharmacists working in general (family) practices substantially reduced the frequency of clinically important medication errors; this reduction is highly likely to translate into fewer preventable adverse drug events in primary care. Therefore the PINCER trial shows that pharmacists working in a primary care setting can provide a cost-effective intervention that should reduce patient harm.[8,9] The detailed trial methods and main findings have been reported elsewhere.[8–10] In summary, the intervention involved identifying patients potentially at risk of harm from hazardous medicines management using Quest Browser software (The Computer Room, Nottingham, UK) to search general practice electronic patient records. The Quest Browser searches were based on 10 outcome measures relating to contraindicated prescribing, inadequate monitoring and inappropriate dosing of medication (see Box 1). Seventy-two general practices were recruited from an 80 km radius around Manchester and Nottingham in the UK and were randomised to receive either simple feedback (36 practices) or pharmacist intervention (36 practices).

**Box 1 Outcome measures (OM) used to identify patients at risk from hazardous medicines management**

Contraindicated prescribing

OM1 Patients with a history of peptic ulcer who have been prescribed a non-selective non-steroidal anti-inflammatory drug without co-prescription of a proton-pump inhibitor
OM2 Patients with asthma who had been prescribed a b-blocker
OM 4 Women with a past medical history of venous or arterial thrombosis who had been prescribed the combined oral contraceptive pill

Inadequate monitoring

OM 3 Patients aged 75 years and older who have been prescribed an angiotensin-converting enzyme inhibitor or a loop diuretic long-term who had not had a computer-recorded check of their renal function and electrolytes in the previous 15 months
OM 5 Patients receiving methotrexate for at least 3 months who had not had a full blood count recorded, or liver function test, in the previous 3 months
OM 6 Patients receiving warfarin for at least 3 months who had not had a recorded check of their international normalised ratio in the previous 12 weeks
OM 7 Patients receiving lithium for at least 3 months who had not had a recorded check of their lithium concentrations in the previous 3 months
OM 8 Patients receiving amiodarone for at least 6 months who had not had a thyroid function test in the previous 6 months

Dosing problems

OM 9 Patients receiving prescriptions of methotrexate without instructions that the drug should be taken every week
OM 10 Patients receiving prescriptions of amiodarone for at least 1 month who are receiving a dose of more than 200 mg per day

Practices receiving simple feedback were given paper copies of the Quest Browser search results and evidence-based summaries to support each outcome measure. These practices did not receive support from a pharmacist as part of the trial. In the pharmacist-intervention arm, six pharmacists with varied backgrounds[11] worked for 2 days per week for up to 12 weeks (i.e. one pharmacist per practice). At the start of their work in each practice the pharmacists met with members of the practice team to discuss the Quest Browser-generated feedback on patients with potentially hazardous medicines management. Where possible this meeting included all general practitioners (GPs) from the practice, at least one nurse, one receptionist and the practice manager. Before the meeting, practice staff were given a summary of the Quest Browser-generated feedback and copies of evidence-based summaries which supported each outcome measure. During the meeting, pharmacists used the principles of educational outreach[12–14] to communicate important messages about why the medicines management was hazardous while also taking account of human error theory[15] and using root-cause analysis techniques, as appropriate.[16] Following this meeting the pharmacists worked closely with a designated liaison person in each practice to help improve systems of work to prevent future medicines management problems. After 6–8 weeks pharmacists held follow-up meetings with practice staff to review progress. To resolve existing medicines management problems pharmacists also reviewed each case of potentially hazardous medicines management identified by the Quest Browser searches. Pharmacists used their clinical expertise to determine whether the patients identified were at clinical risk of harm, and recommended interventions to reduce the risk of harm to patients. Interventions were communicated to GPs using a handwritten form (the case-specific record; see Box 2). Where interventions were accepted by GPs, pharmacists and other members of the practice staff implemented the changes. Where interventions were not accepted GPs either continued current care or recommended their own changes to patients' treatments.

**Box 2 Forms completed by pharmacists when reviewing individual patient cases**

Form 1: the ‘summary record recorded the following data:

- patient ID (numerical code)
- patient age and gender
- GP's initials
- outcome measure (numerical code) (see Box 1)
- numerical code describing pharmacist's judgement of why a patient was identified by the Quest Browser search (patient at risk of harm, coding error, information available in medical records but not coded, other reason)
- numerical code describing pharmacist's interventions and actions completed
- pharmacist's estimate of the time they spent reviewing each case and implementing actions
- pharmacist's initials and date record completed.

Form 2: the case-specific record recorded the following data

- detailed notes describing each patient's medication problem
- description of interventions which the pharmacist recommended
- GP's comments about the recommended interventions (not completed in all cases)
- description of actions taken to resolve the medication problem
- summary of how the patient was contacted.

## 

This paper aims to describe the patient-specific interventions recommended by pharmacists implementing the PINCER trial intervention, how much time pharmacists spent on these interventions and the percentage of interventions accepted by GPs. This will help pharmacists and commissioners of services understand how the patient-specific work of pharmacists in primary care can help reduce the risk of preventable adverse drug events.

## Methods

Ethical approval for the PINCER trial was obtained from the Nottingham 2 Research Ethics Committee (reference number 05/Q2404/26) on 15 March 2005.

### Data collection

Six PINCER pharmacists manually completed two record forms: a summary record (Form 1) and a case-specific record (Form 2) (see Box 2). These forms recorded the pharmacists' activity for each case that they reviewed while working in intervention practices. Additionally, pharmacists were also asked to record on Form 1 whether they judged a patient to have been identified by the Quest Browser searches (i) because they were at clinical risk of harm, (ii) because there was an error in the medical record (coding error), (iii) because information was available in the medical records but not coded or (iv) for another reason (pharmacists were not asked to specify the other reason).

### Data entry

Data from the summary record (Form 1) were entered into SPSS version 15[17] and data entry was independently double-checked for accuracy. Discrepancies were noted and corrected by referring to the summary record in 553 (27%) cases. Where interventions and actions completed were coded as ‘other action’, case-specific records (Form 2) were reviewed (597/2038; 29%) and the actions recoded as one of an additional 53 types of intervention or action completed. These data were single-entered into SPSS version 15.[17] Data were validated by checking that actions recommended or completed were appropriately coded for each outcome measure (discrepancies were noted in 15 (2%) entries and corrected by referring back to the case-specific record). Duplicate data were noted for eight cases and these duplicate entries were removed.

Interventions recommended by pharmacists were compared with actions completed within the practice for each case and coded to indicate whether interventions had been accepted, rejected, were still to be completed, an alternative or additional action had been taken, or the outcome was unknown. Data entry was double-checked and discrepancies noted and corrected in 74 (4%) cases.

### Data analysis

Data were summarised using percentages for categorical data (95% confidence interval (CI)), and means (± standard deviation) for normally distributed data and medians (interquartile range) for non-normally distributed data.

## Results

### Patient characteristics

A total of 1946 patients (from 228 748 patients registered in 36 practices) were identified as at risk from 2038 cases of potentially hazardous medicines management. Ninety-five per cent (95% CI 95, 95; 1854/1946) of patients were identified by one outcome measure, 5% (95% CI 4, 6; 92/1946) were identified by two outcome measures and none were identified by more than two outcome measures. Fifty-nine per cent (95% CI 57, 61; 1149/1946) of patients were female and the mean age was 68.2 years (standard deviation ±16.3 years).

### Pharmacists' judgements of why patients were identified by Quest Browser searches

Pharmacists judged that 1463/2026 (72%; 95% CI 70, 74) cases were clinically at risk (data were missing for 12 cases which pharmacists did not have time to review). The reasons why cases identified by the Quest Browser searches were judged not to be at clinical risk were: (i) because the necessary information was available, but not coded on the computer (43/2026; 2%; 95% CI 2, 3) (for example, blood results were received in letters and scanned into the computer, but not coded into the results section of a patient's electronic record), (ii) because the information had been coded incorrectly on the computer (66/2026; 3%; 95% CI 3, 4) and (iii) for other ‘unspecified’ reasons (461/2026; 23%; 95% CI 16, 30) (Table 1).

**Table 1:** Number (%) of cases judged to be at clinical risk by outcome measure

Outcome measure	Number (%) cases identified (*n* = 2038)	Number cases at clinical risk (% of cases identified for each outcome measure)	Median (interquartile range) time taken for a pharmacist to manage each case (min)	Total pharmacist intervention time (h)
1 Non-steroidal anti-inflammatory drug and peptic ulcer	89 (5)	80 (90)	20 (15, 30)	38.5
2 Asthma and β-blocker	535 (26)	433 (81)	30 (15, 45)	273.2
3 Angiotensin-converting enzyme inhibitor/diuretic and laboratory test	561 (28)	526 (94)	15 (10, 30)	198.6
4 Arterial or venous thrombosis and combined oral contraceptive	5 (0)	4 (80)	10 (10, 40)	1.8
5 Methotrexate and full blood count or liver function test	181 (9)	105 (58)	17.5 (10, 25)	58.6
6 Warfarin and INR	213 (11)	50 (24)	15 (10, 20)	28.5
7 Lithium and lithium levels	99 (5)	74 (75)	15 (10, 30)	33.6
8 Amiodarone and thyroid-function tests	118 (6)	112 (95)	20 (15, 30)	50.4
9 Methotrexate and weekly dosage	228 (11)	73 (32)	10 (5,15)	45.7
10 Amiodarone and daily dosage	9 (0)	6 (67)	20 (10, 40)	3.8
All cases	**2038 (100)**	**1463 (72)**	**20 (10, 30)**	**732.7**

INR, International Normalised Ratio.

The percentage of at-risk cases varied markedly between outcome measures (Table 1), with 90% or more cases considered at risk for outcome measures 1, 3 and 8, whereas fewer than 40% of cases were considered to be at risk for outcome measures 6 and 9 (see Table 1).

### Estimated time taken for pharmacists to review cases, recommend interventions and complete agreed actions

Pharmacists estimated that they took a median of 20 min (interquartile range 10, 30) to review each case, recommend interventions and complete agreed actions. The median estimated time taken varied between outcome measures from 10 min (to manage cases associated with oral contraceptive pills and arterial or venous thrombosis and inappropriate dosing of methotrexate) to 30 min (for cases associated with asthma and β-blockers) (Table 1). The total estimated time spent assessing cases, making recommendations and completing agreed actions was 732.7 h (97.7 working days); equivalent to 2.7 working days per practice (based on a standard NHS pharmacist contract of 7.5 h per day). This varied from a median of 1.2 days (range 0.5–1.6 days) for small practices (list size <2500 patients), a median of 1.3 days (range 0.6–3.2 days) for medium practices (list size 2500–6000 patients) and a median of 3.3 days (range 1.2–8.2 days) for large practices (list size >6000 patients).

### Number of interventions recommended by the pharmacists

Pharmacists recommended 2105 interventions to help resolve medication problems in 1516/2038 cases (74%; 95% CI 73, 76) identified by the Quest Browser searches. In 1073 cases (53%; 95% CI 50, 55) one recommendation was made, in 335 cases (16%; 95% CI 16, 17) two recommendations were made, in 70 cases (3%; 95% CI 3, 4) three recommendations were made and in 38 cases (2%; 95% CI 1, 2) four recommendations were made. In those judged to be at risk of clinical harm, overall 1782 interventions were recommended in 1282/1463 cases (88%; 95% CI 86, 89).

### Actions taken to reduce the risk to patients from hazardous medicines management

Overall, 1685 actions were taken in 1246/2038 cases (61%; 95% CI 59, 63) identified by the Quest Browser searches. Some 1383/2105 (66%; 95% CI 64, 68) of the interventions recommended by pharmacists were completed. Where pharmacists considered cases to be at risk of clinical harm 1420 actions were undertaken in 1066/1463 cases (73%; 95% CI 71, 75). In these at-risk cases 1165/1782 interventions (65%; 95% CI 63, 68) recommended by pharmacists were completed. In addition to the interventions recommended by pharmacists, GPs took additional or alternative actions. Also, some interventions were accepted by GPs, but the pharmacists documented that they had not been completed when the pharmacist finished working in the practice. For example, a GP agreed that a patient's β-blocker prescription should be reviewed, but this review had not happened when the pharmacist left the practice. In other cases it was unknown whether the intervention had been accepted because the pharmacist had not documented on the summary record that the intervention was completed or rejected (Table 2).

**Table 2:** Number of interventions recommended by PINCER pharmacists, and subsequent actions taken in response

		Number of interventions (% of interventions recommended)	
		All cases	Cases considered to be at clinical risk
Interventions recommended by pharmacists		2105	1782
Actions taken by pharmacists and practice staff		1685	1420
Outcome of interventions recommended by pharmacists	Completed	1383 (66)	1165 (65)
	Rejected by GP or patient	441 (21)	357 (20)
	Unknown whether completed or rejected	177 (8)	160 (9)
	Accepted by GP, but not completed at end of placement	104 (5)	99 (6)
GP took an alternative action to that recommended by the pharmacist (% of actions taken)		201 (12)	159 (11)

### Interventions recommended for contraindicated prescribing problems

Pharmacists recommended 804 interventions in 515/629 cases (82%; 95% CI 79, 85) of contraindicated prescribing associated with β-blockers, non-steroidal anti-inflammatory drugs (NSAIDs) and combined oral contraceptives. Actions were taken to help resolve problems with contraindicated prescribing in 370/629 cases (59%; 95% CI 55, 63). Ninety per cent of pharmacists' recommendations to update electronic patient records with properly coded test results or diagnoses were accepted; half of pharmacists' recommendations to review contraindicated medications or monitor patients more closely were accepted and a third of recommendations to prescribe protective medication or counsel patients were accepted. Adding screen messages to the electronic patient records to remind GPs to undertake these recommendations were completed in more cases than were recommended by pharmacists (see Table 3) because GPs suggested adding these reminders after reading pharmacists' interventions.

**Table 3:** The interventions recommended, and actions taken, in cases of contraindicated prescribing

Description of interventions recommended by pharmacists	No (%) of interventions (*n* = 804)	Description of actions taken by pharmacists and practice staff	No (%) of actions (*n* = 512)
**Recommend review contraindicated medication**	**337 (42)**	**Contraindicated medication reviewed**	**175 (34)**
Review, stop or wean β-blocker, or change to bisoprolol	279 (35)	β-Blocker stopped or weaned off	68 (13)
		β-Blocker to be reviewed	36 (7)
		β-Blocker changed to bisoprolol	23 (4)
Contact consultant to query β-blocker	19 (2)	β-Blocker queried with consultant	15 (3)
Stop NSAID or combined oral contraceptive	39 (5)	NSAID or combined oral contraceptive stopped	29 (6)
		Other	4 (1)
**Recommend monitoring**	**188 (23)**	**Monitoring need accepted**	**95 (19)**
Recommend asthma monitoring	179 (22)	Asthma review booked	68 (13)
Recommend other monitoring (blood pressure or medication reviews)	9 (1)	Other monitoring completed (blood pressure or medication review)	27 (5)
**Recommend amend patient's medical record**	**106 (13)**	**Patient's medical record amended**	**100 (20)**
Correct coding error	66 (8)	Correct coding error	69 (13)
Add codes (e.g. blood test result, asthma resolved to history or diagnosis detail to problem screen)	24 (3)	Add codes (e.g. blood test)	14 (3)
Request confirmation of diagnosis	20 (2)	Diagnosis confirmed	13 (3)
**Recommend add screen messages to medical record as reminders**	**59 (7)**	**Screen messages added to medical record**	**107 (21)**
Add screen message advising avoid NSAIDs	25 (3)	Screen message added advising avoid NSAIDs	29 (6)
Add screen message reminding to monitor asthma with β-blocker	23 (3)	Screen message added reminding to monitor asthma with β-blocker	46 (9)
Add other screen messages	11 (1)	Other screen messages added	32 (6)
**Recommend patient counselling**	**54 (7)**	**Patient counselling completed**	**18 (4)**
Counsel patient regarding risks of taking medication	54 (7)	Patient counselled regarding risks of taking medication	18 (4)
**Recommend review protective medication**	**33 (4)**	**Protective medication reviewed**	**12 (2)**
Add proton-pump inhibitor	29 (4)	Add proton-pump inhibitor	11 (2)
Other	4 (0)	Other	1 (0)
**Recommend other changes (not directly related to the indicator)**	**27 (3)**	**Other changes made (not directly related to the indicator)**	**5 (1)**

NSAID, non-steroidal anti-inflammatory drug.

### Interventions recommended for monitoring problems

Pharmacists recommended 1171 interventions in 878/1172 cases (75%; 95% CI 73, 77) of inadequate monitoring in patients taking diuretics, angiotensin-converting enzyme inhibitors, lithium, amiodarone, warfarin and methotrexate. Some 1073 actions were taken to help resolve problems with inadequate monitoring in 796/1172 cases (68%; 95% CI 65, 71). Pharmacists' recommendations to enter existing test results, arrange appointments for patients to have blood tests, contact secondary care to obtain existing test results and ask specialist clinics to amend the general practice code used for lab-linking to ensure that test results are automatically received were accepted in 89% of cases (885/997; 95% CI 87, 91). Recommendations to use searches of computer systems to highlight patients overdue for monitoring, set up screen reminders to ensure patients have regular monitoring, and clarify and record who is responsible for monitoring patients were completed in more cases than they were recommended because GPs recommended running these searches after reading pharmacists' interventions (see Table 4).

**Table 4:** The interventions recommended, and actions taken, in cases of inadequate monitoring

Description of interventions recommended by pharmacists	No (%) of interventions (*n* = 1171)	Description of actions taken by pharmacists and practice staff	No (%) of actions (*n* = 1065)
**Getting and recording test results/monitoring**	**997 (85)**	**Getting and recording test results/monitoring**	**885 (83)**
Arrange blood tests	703 (60)	Blood tests arranged	614 (58)
Read code blood test results	148 (13)	Blood test results read coded	132 (12)
Obtain blood test results from secondary care or self-monitoring patient and read code	84 (7)	Blood test results obtained from secondary care and self-monitoring patients and read coded	87 (8)
Contact warfarin clinic and request regular results via lablink	55 (5)	Warfarin clinic changed general practice code to ensure ‘lab-link’ results received	43 (4)
Other interventions recommended relating to monitoring	7 (1)	Other actions taken related to monitoring	8 (1)
		Patient specific reasons why monitoring not completed	12 (1)*
**Recommend ways to identify patients needing monitoring**	**150 (13)**	**Actions completed to help identify patients needing monitoring**	**160 (15)**
Monitor exceptions with search template	55 (5)	Monitor exceptions with search template	51 (5)
Review and record monitoring arrangements on electronic patient record	49 (4)	Monitoring arrangements confirmed and documented on electronic patient record	57 (5)
Screen message to monitor bloods 3-monthly	46 (4)	Screen message re: bloods added	52 (5)
**Changes to medication recommended (unrelated to indicator)**	**17 (1)**	**Changes to medication made (unrelated to the indicator)**	**12 (1)**
**Changes to medication recommended due to deranged blood test results**	**7 (1)**	**Medication changes completed due to deranged blood test results**	**8 (1)**

* Includes nine patients who declined bloods, two patients who could not be contacted and one patient who could not be bled.

### Interventions recommended for dosing problems

Pharmacists recommended 130 interventions in 123/237 cases (52%; 95% CI 46, 58) of potential dosing problems in patients taking methotrexate and amiodarone. Eighty-six actions were taken to help resolve problems with inadequate monitoring in 80/237 cases (34%; 95% CI 28, 40). Pharmacists' recommendations to alter the quantity or strength of methotrexate supplied were accepted in all five cases, to amend dosage instructions of methotrexate or amiodarone were accepted in 64% of cases (67/104; 95% CI 55, 74) and to start or increase the dose of folic acid were accepted in 75% of cases (6/8; 95% CI 45, 101) (see Table 5).

**Table 5:** The interventions recommended, and actions taken, in cases with dosing problems

Description of interventions recommended by pharmacists	No (%) of interventions (*n* = 130)	Description of actions taken by pharmacists and practice staff	No (%) of actions (*n* = 86)
**Recommend changes to prescription**	**106 (82)**	**Recommend changes to prescription**	**69 (80)**
Alter dosage instructions for methotrexate or amiodarone	104 (80)	Dosage instructions for methotrexate or amiodarone altered	67 (78)
Other	2 (2)	Other	2 (2)
**Other actions (not directly related to the indicator)**	**24 (18)**	**Other actions (not directly related to the indicator)**	**17 (20)**
Recommend prescribe, or increase dose of, folic acid	8 (6)	Folic acid prescribed, or dose increased	6 (7)
Other interventions related to monitoring methotrexate or amiodarone	7 (5)	Other actions taken related to monitoring methotrexate or amiodarone	3 (3)
Recommend change strength of methotrexate tablets or reduce quantity supplied	5 (4)	Methotrexate strength changed or quantity supplied reduced	5 (6)
Other action (unspecified)	4 (3)	Other action (unspecified)	3 (3)

## Discussion

Analysis of the pharmacist-recorded data on individual cases of potentially hazardous medicines management identified by the Quest Browser searches has shown that pharmacists judged 72% of cases to be at clinical risk of harm. Pharmacists recommended interventions to help improve the safety of medicines management in 74% of cases identified and 66% of their interventions were accepted by GPs and completed. Additional or alternative actions to improve the safety of medicines management were taken by GPs, meaning that actions were completed in 61% of cases identified by the Quest Browser searches. Pharmacists estimated that they spent a median of 20 min reviewing each case, recommending interventions and completing actions. Pharmacists recommended a broad range of interventions to help reduce the risk of harm to individual patients. Many of these recommendations would have implications for the future management of these patients and, in some cases, other patients within the practices.

### Strengths and limitations

The PINCER trial was a large cluster randomised controlled trial which clearly demonstrated the effectiveness of a pharmacist-led IT-enabled intervention in reducing the risk to patients from hazardous medicines management. The strengths and limitations of the trial have been discussed in detail elsewhere.[8,9,11,18]

During their work on the PINCER trial, pharmacists recorded data on individual cases of potentially hazardous medicines management. As part of this data recording, pharmacists judged whether patients identified by the Quest Browser searches were at risk of clinical harm. These judgements were based on the clinical expertise of one pharmacist in each case and other healthcare professionals may not agree with their judgements. Pharmacists did not keep detailed records of the basis for their judgements; therefore, it is not possible to further identify their ‘unspecified reasons’ for not judging patients to be at clinical risk.

Additionally, pharmacists' recommendations to improve medicines management were not peer reviewed for appropriateness or missed opportunities to intervene. Therefore, it is possible that some interventions were rejected by GPs because they were inappropriate or that opportunities to improve medicines management were missed.

The data recorded by the pharmacists help us understand how they approach individual cases of hazardous medicines management in order to help protect patients from harm. These data also help us predict which interventions are most likely to be accepted by general practice staff. These data do not, however, help us understand why some recommendations were not accepted.

Pharmacists estimated the time they spent reviewing, recommending interventions and completing actions for individual cases of hazardous medicines management. These times may under- or over-estimate the actual time spent by the pharmacists on individual patient cases and do not indicate how long was spent on each specific task. Pharmacists' estimates of time spent reviewing individual cases indicate that most time was spent on other duties such as meeting with practice staff and implementing new systems of working in the practices; however, pharmacists did not record the time they spent on these activities. Additionally, these roles are not detailed in the pharmacist-recorded data and this limits the usefulness of these data in terms of understanding the role of the pharmacist in the PINCER trial. However, additional qualitative data collected as part of the PINCER trial[18] does provide better understanding of this extended role.

### Usefulness of outcome measures for identifying patients at clinical risk of harm

Pharmacists judged that three-quarters of cases identified by Quest Browser searches were at clinical risk of harm, suggesting that such outcome measures are a useful way of targeting pharmacists' activity. However, the proportion of patients considered at clinical risk of harm varied greatly between outcome measures. Ninety to ninety-five per cent of patients identified by outcome measures 1, 3 and 8 (relating to contraindicated prescribing of NSAIDs and inadequate monitoring with diuretics, angiotensin-converting enzyme inhibitors and amiodarone) were judged to be at clinical risk. In contrast, one-quarter of patients identified by outcome measure 6 (related to inadequate monitoring of warfarin) were judged to be at clinical risk. However, 54% (114/213) of these were registered in one practice that had a separate recording system for patients' International Normalised Ratios (INRs), which was not identified by the Quest Browser searches. By excluding these data, half of patients were considered to be at clinical risk by the pharmacists. In outcome measure 9 (related to the inappropriate dosing of methotrexate) the IT-enabled searches were unable to reliably identify patients without a weekly instruction. This meant that only 32% of patients identified as having no instruction to take their methotrexate once weekly were judged to be at clinical risk. This is an important problem which needs to be resolved before IT-enabled searches can be used to identify patients at risk from inappropriate dosing of medication.

### Pharmacists' interventions

Pharmacists made recommendations to improve medicines management in 74% of the cases identified and in 88% of cases where they considered patients to be at clinical risk. This is consistent with Zermansky *et al*. where pharmacists recommended interventions for 75% of general practice patients.[19] In the PINCER trial, 66% of interventions recommended by a pharmacist were completed, compared to 56% of pharmacists' interventions recommended in the Zermansky *et al*. study of medication reviews in a nursing home setting.[20,21] This suggests that pharmacists' interventions were broadly acceptable to the GPs. This finding is supported by a qualitative evaluation of the PINCER intervention, which demonstrated that the trial intervention had face validity and was acceptable to GPs and their teams.[18]

In the intervention arm of the PINCER trial one-fifth of pharmacists' recommendations were not accepted by GPs or patients. However, even when pharmacists' interventions were not acted on (around 12% of cases) GPs took additional or alternative actions to those recommended by the pharmacists. This suggests that, by highlighting potential problems with patients' medication, pharmacists stimulated GPs to make changes to their management of these patients. This effect was also seen in the nursing home study by Zermansky *et al*. where GPs took additional or alternative actions to those recommended by pharmacists in 5% of cases.[20,21]

In the PINCER trial the number of interventions recommended per case varied, with half of cases having only one intervention. Where multiple recommendations were made they could be of the form ‘I recommend action A, but if this is not suitable/accepted, then please consider action B’. This would be recorded as two separate interventions but it would not be possible for a GP to accept both interventions. This may in part explain why only one-third of pharmacists' interventions were accepted in outcome measure 2; pharmacists recommended two or more interventions in 45% of the cases where they made recommendations. However it is not possible to state with certainty that all of these recommendations were mutually exclusive. Future studies of pharmacists' interventions should assess the form in which recommendations are made when calculating levels of acceptance.

PINCER trial pharmacists' interventions were targeted to the outcome measure being addressed. These interventions were broadly applicable across the outcome measures in the three groups, however: contraindicated prescribing, inadequate monitoring and dosing problems. This suggests that these interventions could be broadly applicable to a wider range of clinical situations related to these groups of outcome measures. Many of these interventions were not predicted before the start of the study. Instead, they developed from pharmacists' experience of working with the GPs and learning from each other. This highlights the importance of providing a forum where pharmacists can learn from each other when engaging in new roles. This forum was created in the PINCER trial through monthly group meetings between the pharmacists and the trial manager.

As might be predicted from the broad range of different recommendations made, the time spent resolving problems in each case varied widely (from 0 to 180 min). However, pharmacists reported spending a median of 20 min on each case. This included the time needed to assess each case, make recommendations and implement any agreed changes. This is consistent with the average time taken to conduct medication reviews in a previous study in primary care,[19] but is likely to be lower in terms of overall time spent because there was little face-to-face contact between patients and pharmacists in the PINCER trial. Instead, medication reviews were largely restricted to medical records because this was how the practices preferred to work (instead, GPs had face-to-face meetings with patients).

The median time pharmacists spent on each case varied depending on the outcome measure and practice list size. Although outcome measure 2 (β-blockers in asthma) had the lowest percentage of completed actions it was the most time-consuming of the outcome measures. Before the study began we predicted that outcome measure 2 would be the most difficult for pharmacists to address because of the complexity of assessing the cases and lack of clear guidelines on how to manage patients. This is reflected in the time taken to review the cases and percentage of cases where actions were completed.

Pharmacists required less time to review cases and recommend interventions than anticipated at the start of the trial. This suggests that, with adequate training, it may be possible to ‘roll out’ the pharmacist intervention using existing pharmacist staff within general practices.

## Conclusions

Analysis of pharmacist-recorded data from the PINCER trial suggests that the outcome measures usefully targeted pharmacists' activities towards patients who may have been at risk of hazardous medicines management and that GPs were largely supportive of the approach taken in the trial.

This analysis has also highlighted areas which were challenging for pharmacists to influence, such as the prescription of β-blockers to patients with asthma and cardiovascular disease. The analysis has also identified some useful interventions which could be applied to a wide range of clinical scenarios. In addition, it would seem that the activities undertaken by the pharmacists were largely appropriate to their level of training, although it may be possible to identify some activities that could be undertaken by less qualified staff, such as pharmacy technicians, once pharmacists have performed a clinical review of individual cases.

## Declarations

### Conflict of interest

The Author(s) declare(s) that they have no conflicts of interest to disclose.

### Funding

This work was supported by the Patient Safety Research Portfolio UK (grant number PS024; current controlled trials number ISRCTN21785299).
